# The Use of a Bayesian Hierarchy to Develop and Validate a Co-Morbidity Score to Predict Mortality for Linked Primary and Secondary Care Data from the NHS in England

**DOI:** 10.1371/journal.pone.0165507

**Published:** 2016-10-27

**Authors:** Colin J. Crooks, Tim R. Card, Joe West

**Affiliations:** 1 Division of Epidemiology and Public Health, Clinical Sciences Building, University of Nottingham, Nottingham City Hospital, Nottingham, Nottinghamshire, United Kingdom; 2 National Institute for Health Research (NIHR) Biomedical Research Unit in Gastrointestinal and Liver Diseases at Nottingham University Hospitals NHS Trust and the University of Nottingham, Queens Medical Centre Campus, E Floor West Block, Derby Road, Nottingham, Nottinghamshire, United Kingdom; Universidade do Algarve, PORTUGAL

## Abstract

**Background:**

We have assessed whether the linkage between routine primary and secondary care records provided an opportunity to develop an improved population based co-morbidity score with the combined information on co-morbidities from both health care settings.

**Methods:**

We extracted all people older than 20 years at the start of 2005 within the linkage between the Hospital Episodes Statistics, Clinical Practice Research Datalink, and Office for National Statistics death register in England. A random 50% sample was used to identify relevant diagnostic codes using a Bayesian hierarchy to share information between similar Read and ICD 10 code groupings. Internal validation of the score was performed in the remaining 50% and discrimination was assessed using Harrell’s C statistic. Comparisons were made over time, age, and consultation rate with the Charlson and Elixhauser indexes.

**Results:**

657,264 people were followed up from the 1st January 2005. 98 groupings of codes were derived from the Bayesian hierarchy, and 37 had an adjusted weighting of greater than zero in the Cox proportional hazards model. 11 of these groupings had a different weighting dependent on whether they were coded from hospital or primary care. The C statistic reduced from 0.88 (95% confidence interval 0.88–0.88) in the first year of follow up, to 0.85 (0.85–0.85) including all 5 years. When we stratified the linked score by consultation rate the association with mortality remained consistent, but there was a significant interaction with age, with improved discrimination and fit in those under 50 years old (C = 0.85, 0.83–0.87) compared to the Charlson (C = 0.79, 0.77–0.82) or Elixhauser index (C = 0.81, 0.79–0.83).

**Conclusions:**

The use of linked population based primary and secondary care data developed a co-morbidity score that had improved discrimination, particularly in younger age groups, and had a greater effect when adjusting for co-morbidity than existing scores.

## Introduction

There is a critical lack of a co-morbidity index derived for the general population outside of secondary care cohorts, as no co-morbidity index has been derived directly for linked primary and secondary care data. A measure of co-morbidity is essential to adjust disease outcomes for confounding by coexisting chronic illness. The best validated method to date is the secondary care derived Charlson index, developed using hospital chart data of medical inpatients to predict mortality in the 1-year post discharge, and validated in a cohort of breast cancer inpatients in 1987[1]. An adaptation of the Charlson index has been translated to the Read/OXMIS code system for primary care, but it did not reassess which diseases to include or their weightings [2]. Management of various diseases has changed greatly over the last two decades and a diagnosis might have a different contemporary association with mortality than it did in 1987. This was partly confirmed in another study which found that the Charlson index weightings did need updating, but the researchers only used hospital data and did not assess whether additional diagnoses outside of the Charlson index might now be relevant [3].

Other frequently used measures such as the Elixhauser [4] and Chronic Disease Score [5] use a wider range of co-morbidity. However, they were designed to predict hospital costs, length of stay and short term 30-day mortality as outcomes. These outcomes might conflict with each other, for example Elixhauser et al. reported that depression, obesity, and hypothyroidism increased length of stay and hospital costs, yet found these diagnoses were actually protective for in hospital mortality. Combining these outcomes can therefore confuse the utility of these scores when used outside a health economics setting to predict survival.

Another consequence of existing scores like the Charlson index being derived from medical inpatient data is that the medical co-morbidities commonly found in this more elderly population predominate, whilst other co-morbidities that might be relevant in a younger population can be overlooked, such as mental health [6]. An unselected population based cohort could avoid this problem whilst also assessing whether co-morbidity recorded during hospital admissions has different mortality associations compared to co-morbidity recorded in the community by a general practitioner.

We have therefore aimed to develop a contemporary co-morbidity score in a population based cohort from Clinical Practice Research Datalink using linked primary and secondary care diagnoses. To adjust for multiple testing and potential group effects we used Bayesian data mining techniques we have previously published [7].

## Objective and Aims

To develop a co-morbidity score within linked primary care and secondary care data that utilises ICD 10 and Read codes to predict one-year mortality.

1. To identify potential codes for categories of diagnostic ICD 10 and Read codes that are associated with one-year survival.
2. To test which of these categories predict survival when adjusted in a model using Cox proportional hazards modelling.
3. To validate the prognostic model in a validation sample and test its discriminative ability at different ages, follow up times, and calendar years.
4. To assess the ability of the score to adjust for confounding in a chronic and an acute disease with known mortality risks and compare this with other similar scores.

## Materials and Methods

### Data

A cohort study was designed using linked longitudinal data from the English Hospital Episodes Statistics (HES) data, Clinical Practice Research Datalink (CPRD) and Office of National Statistics (ONS) death register. This data linkage records all primary care events, hospital admissions, and causes of death from 1st April 1997 for 3% of the English population [8]. Because of the comprehensive English primary care system, the population registered to the CPRD is representative of the general English population [9]. The data sources are subject to quality checks and a practice’s data is only used when it is of high enough quality to be used in research. This is referred to as the up to research standard time period and is defined separately for each primary care practice. Regulatory approval for this study was obtained from the Independent Scientific Advisory Committee for the Medicines and Healthcare Products Regulatory Agency database research.

### Study population

The study cohort was defined as all patients registered 1st January 2005 to a primary care practice that contributed to the CPRD until 1st January 2010 and had consented to linkage to HES and ONS. The cohort was followed from the 1st January 2005 to their death or transfer out of a CPRD practice or to 1st January 2006 if earlier. This cohort was randomly divided into two halves. The first half was used to develop the prognostic score, and the second half to internally validate its performance. Follow up was extended to 1st January 2010 as part of the assessment of the score’s performance in the validation.

### Exposure

Diagnostic codes rather than medication codes were used to derive the score, as medications would be a proxy for the direct effect of a disease. For this study we used all diagnostic Read codes from primary care in the CPRD (i.e. chapters A, B, C, D, E, F, G, H, J, K, L, M, N, P, S) in addition to all diagnostic ICD 10 codes from secondary care in HES (chapters A, B, C, D, E, F, G, H, I, J, K, L, M, N, O, Q, S, T). These were extracted prior to two months before the cohort start date (1st November 2004). This two-month exclusion period was chosen to avoid including codes that were palliative or recording a final stage of life.

As there would remain too many codes in the Read and ICD 10 code systems to assess in a single multivariate model and there were likely to be significant correlations between similar codes we first needed to identify candidate groupings of codes to use as potential predictors.

However, grouping codes together incorrectly might hide information about which codes were or were not contributing to the group effect. Therefore, we used a hierarchy within a Bayesian framework that allowed information to be shared at a group level whilst still estimating the effect of individual codes within the group. This hierarchy was defined as the sub chapters in the Read code (the first two digits of the Read code) and the ICD 10 code blocks (the first two digits of the ICD 10 code).

Socioeconomic data was available from ONS data linked to the CPRD. This provided the average quintile of Index of Multiple Deprivation of the registered population at each primary care practice [10].

### Outcome

Dates of all-cause mortality for the whole cohort were extracted from the linked data using the ONS death register. All deaths in England are coded and recorded in the ONS death register from death certificates.

### Score development

#### Data mining in a Bayesian framework

The individual unadjusted associations of each Read and ICD 10 code with survival were initially assessed in a random sample of 50% of the whole CPRD taken in 2005 using a Cox proportional hazards model. The hazard ratios of individual codes were then re-estimated using the Bayesian hierarchy previously described to allow for heterogeneous coding of a particular disease without losing the detail of the individual codes. This method has been previously used and published [7]. We then selected codes whose hazard ratio’s 99% confidence interval excluded 1.2 or whose group’s 99% confidence interval excluded 1.2. A hazard ratio of 1.2 was selected as it was the lower limit used in the Charlson index. The groups that these codes were categorised to were then reviewed manually to assess whether the underlying codes were appropriate to these categories based on the authors’ clinical judgement. Categories for consideration for selection for a new score were then defined from these modified groups.

#### Prognostic model building

A Cox proportional hazards model was constructed containing all the categories defined in the previous section adjusted for age and sex. If there were differences between the Read and ICD 10 codes, this might have been due to the latter being a flag of hospitalisation rather than a specific diagnosis. Therefore, an indicator for hospitalisation in the previous year was also included in the model (excluding the two months prior to the study start date). The category coefficients with a hazard ratio of greater than 1.2 and a 99% confidence interval excluding the null were then translated into weights [11]. The beta coefficient for each category was multiplied by 10 and rounded to the nearest integer for convenience. Goodness of fit was compared between models using Akaike’s Information Criterion and discrimination by the Harrell’s C statistic. The model building process was repeated in bootstrapped samples and the differences in Harrell’s C statistic between the score derived from the samples and the full development score was used to provide an estimate of the optimism introduced [12].

#### Sensitivity analyses

The period before a patient’s death will include coding directly related to the final outcome of death. To avoid including this outcome coding as part of the exposure coding of co-morbidity we conducted two sensitivity analyses. First we rebuilt the model excluding all codes recorded in the 6 months prior to 1st January 2005 (rather than the 2 months in the main analysis). Secondly we rebuilt the model excluding patients with less than 1, 3, or 6 months of follow up.

### Validation

Internal validation was carried out in the remaining 50% of the dataset not yet used in the study. The AIC and Harrell’s C statistic were calculated for the new linked score as well as for the Charlson index and the Elixhauser index for comparison. Confidence intervals for Harrell’s C Statistic were calculated using Roger Newson’s somersD command available for download for Stata (http://www.imperial.ac.uk/nhli/r.newson/stata12/somersd.zip).

#### Stratified analysis of the fit and performance of the linked score in predicting mortality

The performance of the linked score, Charlson index and Elixhauser index in the validation cohort were stratified by age, year of follow up after 2005 (up to 5 years), socioeconomic status, and number of consultations in the previous year. All data management and analyses were carried out using Stata 13 MP16 software (StataCorp. 2014. Stata Statistical Software: Release 13. College Station, TX: StataCorp LP).

#### Performance of the score in adjusting for co-morbidity in diabetes and upper gastrointestinal bleeding

Diabetes is a disease that increases the risk of many other co-morbidities that indirectly reduce survival [13], and upper gastrointestinal bleeding is an acute event in which co-morbidity both predicts its occurrence and its subsequent mortality, both short and long term [14–16]. We therefore used a Cox proportional hazards model adjusted for age and gender to assess the ability of our linked score to adjust for the effect of co-morbidity on survival for both of these diagnoses. For the diabetes analysis we removed the category of diabetes from the calculation of the Charlson index and the linked score, and for upper gastrointestinal bleeding we removed bleed codes, to avoid including the diagnoses twice in the model.

For a chronic disease like diabetes the diagnosis date does not necessarily indicate disease onset, therefore for the analysis we selected all patients with a recording of diabetes prior to November 2004, and followed them up from 1st January 2004 (using the same definition as when defining the Charlson index). This cohort was compared them to all patients without a recording of diabetes prior to November 2004 and followed up from 1st January 2004[17].

In contrast upper gastrointestinal bleeding is an acute event with a defined date of onset. As we have previously developed a method for defining upper gastrointestinal bleeding in the CPRD we used this work to identify all patients with a first recorded bleed in our cohort (2005–2010) [18]. In brief an episode was included if there was a specific code for an incident gastrointestinal bleed in either the primary or secondary care dataset with a concurrent supporting code in the other dataset. We dropped all patients with a less specific diagnosis of gastrointestinal bleeding. We followed all patients up following the first bleed and split the time into the first 60 days for short term mortality, and subsequent to that for long term mortality. Sixty days was chosen as that was the window between primary and secondary care used in our initial definition. Follow up in the cohort still finished on 31st December 2010. For the comparison cohort for upper gastrointestinal bleeding we followed up all patients in our cohort without any upper gastrointestinal bleeding from a random observed date.

## Results

### Study population

657,264 people 20–100 years old were available to be followed up from the 1st January 2005 until 1st January 2006 with 21,672 deaths. After allowing for censored individuals and deaths the population had a mean follow up of 3 years. The age structure of the population was similar to the UK population, as would be expected from a national population database [19].

### Score development

#### Data mining in a Bayesian framework

**Fig 1:**
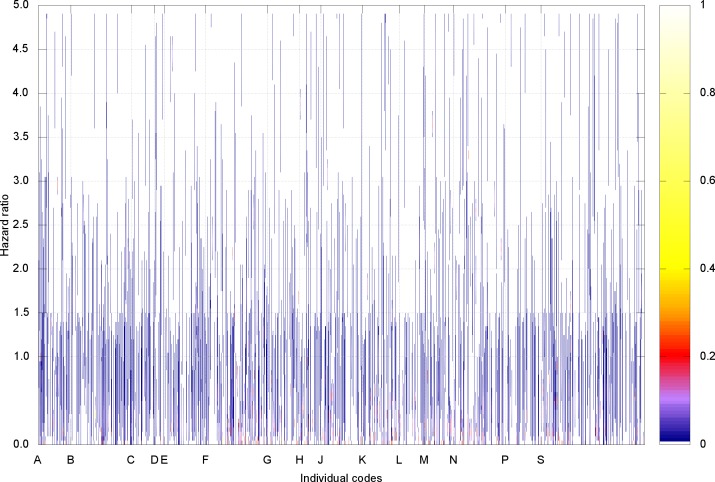
Age and sex adjusted hazard ratio for each individual ICD 10 and Read code. The vertical lines represent the 99% confidence intervals with the colour representing the frequency density as indicated in the colour side bar. The hazard ratios are exponentiated coefficients from the Cox proportional hazards model. For clarity only the broader chapters for codes are labelled (from the mapped Read code chapters).

The age and sex adjusted hazard ratios for each ICD 10 and Read code (13,855 codes in total) in the development population sample (n = 328,628) are shown in Fig 1.

**Fig 2:**
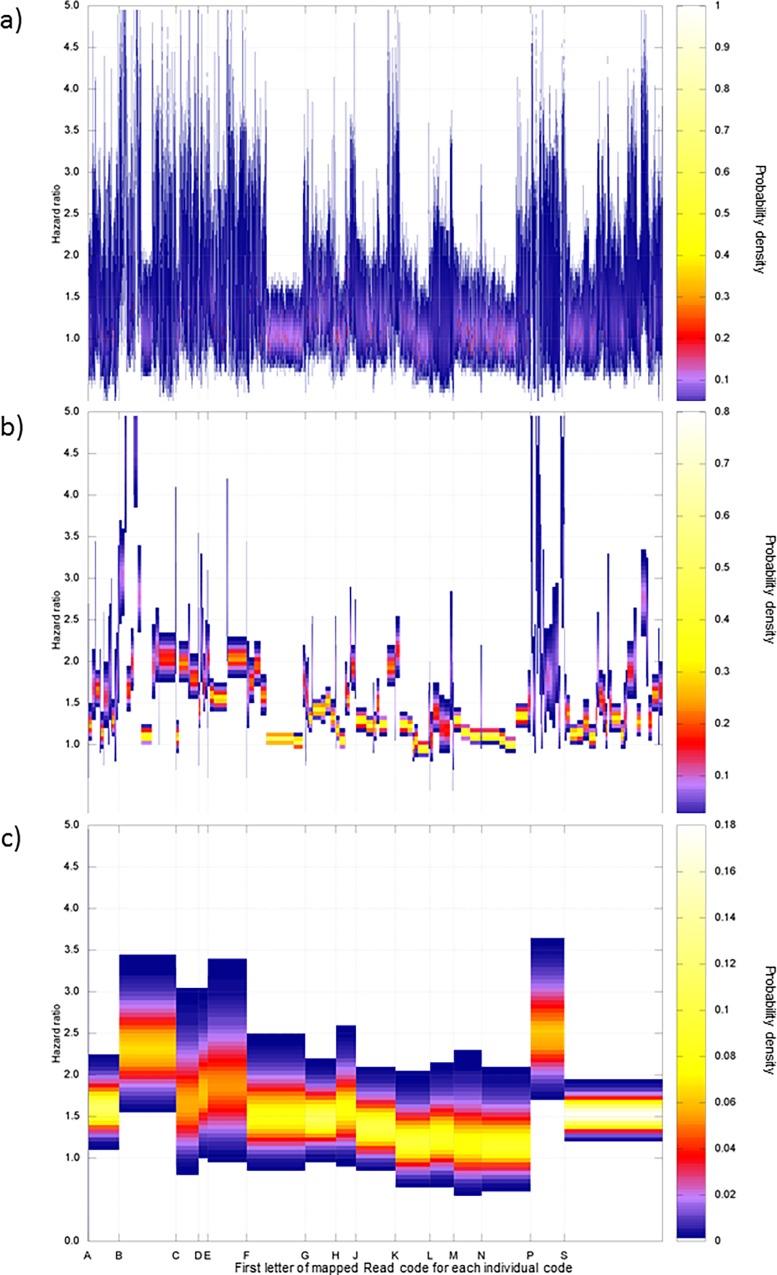
ICD 10 and Read code hazard ratios re-estimated in a Bayesian hierarchy. The three panels are stacked to demonstrate the related probability distributions at each level of the Bayesian hierarchical model with information sharing between codes at both the mapped Read subchapter (leading 2 code digits) and chapter (leading code digit) level: (a) Re-estimated ICD 10 and Read code hazard ratios (b) Mapped Read subchapter group probability distributions. (c) Mapped Read chapter group probability distributions. For all three panels the vertical dimension represents the 99% confidence intervals with the colour representing the probability density as indicated in the colour side bar. For clarity only the broader chapters for codes are labelled on the x axis (from the mapped Read code chapters). A normal prior on the log scale was used for all estimated categories (mean = 0, standard deviation = 1000).

These hazard ratios were re-estimated in the hierarchical Bayesian model using the coding hierarchy to share information between similar codes (Fig 2). Of these re-estimated hazard ratios 644 ICD codes and 801 ICD codes had 99% confidence intervals excluding 1.2. These 1445 codes were grouped by their sub chapters into 96 Read code groups and 97 ICD 10 groups. These produced 98 combined categories after mapping from ICD 10 to Read codes. These groups were then reviewed manually to assess whether the included codes were clinically appropriate to the groups.

#### Prognostic model building

Hazard ratios for the 98 potential categories where derived from a Cox proportional hazards models adjusted for age and gender. Categories derived from Read and ICD 10 codes were added separately. Those categories whose hazard ratio did not significantly differ whether derived from ICD 10 codes or Read codes were then combined. The hazard ratios with 99% confidence intervals excluding the null were then translated into integer weights by rounding the beta coefficient to one decimal place and multiplying by 10 are presented in Table 1 and the codes used have been included as supporting information with this paper (S1 and S2 Tables). The resulting score had a C statistic of 0.882 and an AIC of 244934. Bootstrapping the model building process provided an estimate of the optimism of the C statistic of only +/-0.0005.

**Table 1:** Linked score categories and weights.

	Score for Read codes or combined codes*						Score for ICD 10 codes where significantly different**						
Category label based on Read Sub chapter	Hazard Ratio	95% confidence interval				Score weight	Hazard Ratio	95% confidence interval					Score weight
Neoplasm Histology	1.2	(	1.0	-	1.3)	1							
Malignancy of digestive organs and peritoneum							1.8	(	1.6	-	2.1	)	6
Malignancy of respiratory tract and intrathoracic organs	3.5	(	3.0	-	4.0)	12							
Malignancy of genitourinary organ							1.5	(	1.3	-	1.6	)	4
Malignancy of other and unspecified sites							1.8	(	1.5	-	2.3	)	6
Malignancy of lymphatic and haemopoietic tissue	1.5	(	1.3	-	1.8)	4							
Metastases	2.9	(	2.5	-	3.3)	11							
Non thyroid and non-diabetic endocrine gland disease							1.5	(	1.2	-	1.9	)	4
Diabetes	1.1	(	1.0	-	1.2)	1							
Non deficiency and non-haemolytic anaemias	1.1	(	1.0	-	1.2)	1							
Non-malignant white cell, platelet and splenic disorders	1.2	(	1.0	-	1.4)	2							
Non-organic psychoses	1.3	(	1.2	-	1.5)	3							
Other central nervous system disorders	1.4	(	1.0	-	1.9)	3							
Epilepsy	1.2	(	1.1	-	1.4)	2							
Paralysis	1.2	(	1.1	-	1.4)	2							
Dementia	1.4	(	1.2	-	1.6)	4	2.0	(	1.8	-	2.2	)	7
Parkinson’s disease	1.5	(	1.3	-	1.7)	4							
Spinal disease	1.5	(	1.2	-	2.0)	4							
Multiple Sclerosis							2.4	(	1.7	-	3.5	)	9
Heart conduction disorders	1.2	(	1.1	-	1.2)	2							
Cerebrovascular disease	1.2	(	1.1	-	1.3)	2							
Peripheral vascular disease	1.1	(	1.0	-	1.2)	1	1.4	(	1.2	-	1.5	)	3
Heart failure	1.4	(	1.3	-	1.5)	3							
Chronic obstructive pulmonary disease	1.1	(	1.0	-	1.2)	1	1.5	(	1.3	-	1.6	)	4
Lung disease due to external agents	1.6	(	1.2	-	2.0)	5							
Pleural disease	1.1	(	1.0	-	1.2)	1							
Interstitial lung disease	1.5	(	1.3	-	1.8)	4							
Oesophageal, stomach and duodenal diseases	1.1	(	1.0	-	1.2)	1							
Liver disease	1.4	(	1.1	-	1.7)	3							
Cirrhosis	1.6	(	1.3	-	2.1)	5							
Nephritis, nephrosis and nephrotic syndrome	1.3	(	1.2	-	1.4)	2							
Connective tissue diseases							2.9	(	1.1	-	8.0	)	11
Congenital musculoskeletal deformities							17.3	(	2.8	-	108.2	)	29
Chromosomal anomalies	2.0	(	1.1	-	3.4)	7							
Burns	1.2	(	1.0	-	1.5)	2							
Alcohol or illegal drug use	1.3	(	1.1	-	1.5)	3	1.8	(	1.5	-	2.1	)	6

Linked score categories and weights. Each category is included in the overall score once with the highest weight depending on whether it was identified from Read codes or ICD 10 codes.

*Read or ICD 10 category adjusted for age, sex and all other categories in table.

**If weighting and hazard ratio significantly different (p < 0.005) from the Read or combined hazard ratio the weighting for the ICD 10 code is shown here.

#### Sensitivity analyses

Applying the same weights to ICD 10 and Read codes did not have a large effect on the discrimination of the score (C = 0.880, AIC = 245434), and neither did excluding co-morbidity recorded in the 6 months prior to the start of follow (C statistic = 0.881). As only 4 categories had a weight above 10 we assessed the effect of capping the weights at 10. This did not alter the score’s discrimination (C = 0.882, AIC 244986). Ten percent of deaths (n = 1,100 in the development cohort) occurred within the first 30 days of follow up. Excluding patients with less than 1, 3 or 6 months of follow up from the score development only reduced the discrimination of the score slightly (Table 2).

**Table 2:** Discrimination of the Linked score in the development cohort when developed with patients with less than 1, 3, and 6 months of follow up excluded.

Minimum follow up	Number of people	Total person years	Linked score
≥1	323224	1.08e+08	0.88
≥3	312300	1.07e+08	0.87
≥6	297135	1.05e+08	0.87

### Validation

Compared to the Charlson index when assessing the prediction of death in the second half of the dataset (328,636 people with 10,984 deaths within the first year), the linked score resulted in a better spread across higher values resulting in more stable hazard ratios (S3 Table). Using the score as a categorical or continuous variable did not alter the discrimination (C statistic = 0.879 and 0.878 respectively) but goodness of fit was significantly improved for the former (p < 0.0001 for likelihood ratio test and AIC = 252464 and 252787 respectively). The linked score had significantly improved discrimination and fit compared to the Charlson index and the Elixhauser index (Table 3).

**Table 3:** Performance in the validation cohort for Charlson index, Elixhauser index, and the linked score adjusted for age, gender and recent hospitalisation.

Score	AIC	Harrell’s C statistic	95% Confidence intervals
Elixhauser	254368	0.868	(0.866–0.871)
Charlson index	253724	0.872	(0.869–0.874)
Linked score (categorical)	252460	0.879	(0.876–0.881)
Linked score (continuous)	252755	0.878	(0.875–0.880)

#### Stratified analysis of the fit and performance of the linked score in predicting mortality

There was a significant interaction between age group and the linked score in predicting mortality (likelihood ratio test with nested model without interaction p < 0.0001) and so an age stratified model is presented in Table 4. When stratified by age the discrimination of the linked score was higher than either the Charlson index or the Elixhauser index, particularly in younger age groups. There was also a significant interaction between the linked score and the consultation rate (likelihood ratio test with nested model without interaction p < 0.0001) with some reduction in discrimination for all measure for those who consulted with their general practitioner more than 14 times a year (Table 5). However, the linked score still performed slightly better than the Charlson and Elixhauser index. Finally, when we assessed the performance of the Charlson index during additional years of follow up, the C statistic reduced slightly for each individual year of follow up for the linked score in a similar manner to the Charlson and Elixhauser index (Table 6). When predicting death across the full 5 years of follow up the C statistic for the linked score was 0.85 (0.85–0.85). The linked score had a slightly improved discrimination across most socioeconomic classes (S4 Table), however the addition of socioeconomic data to the model with the linked score produced only a slight improvement in discrimination and was therefore not included in our main analysis.

**Table 4:** Discrimination of the Linked score, Charlson index and Elixhauser index stratified by 10-year age groups in the validation cohort.

Age (years)	N	(%)	Linked score	Charlson index	Elixhauser index
20–29	70140	0.21	0.78 (0.72–0.85)	0.69* (0.63–0.75)	0.71 (0.66–0.77)
30–39	68104	0.21	0.81 (0.76–0.85)	0.73* (0.69–0.78)	0.77 (0.73–0.81)
40–49	46328	0.14	0.81 (0.78–0.84)	0.73* (0.70–0.76)	0.75* (0.72–0.78)
50–59	37433	0.11	0.78 (0.76–0.80)	0.76 (0.74–0.78)	0.75* (0.73–0.77)
60–69	30634	0.09	0.74 (0.72–0.75)	0.73 (0.71–0.74)	0.70* (0.69–0.72)
70–79	32203	0.10	0.70 (0.68–0.71)	0.67* (0.66–0.68)	0.66* (0.65–0.67)
80–89	33687	0.10	0.65 (0.64–0.66)	0.63* (0.62–0.64)	0.63* (0.62–0.64)
≥90	10107	0.03	0.60 (0.59–0.62)	0.58* (0.57–0.59)	0.58* (0.57–0.59)

* Indicates significantly different from linked score C statistic (p<0.05)

**Table 5:** Discrimination of the Linked score, Charlson index and Elixhauser index stratified by primary care consultation rate in the validation cohort.

	N	(%)	Linked score	Charlson index	Elixhauser index
0	69586	0.21	0.88 (0.86–0.89)	0.87* (0.85–0.89)	0.87* (0.85–0.89)
1	35081	0.11	0.91 (0.89–0.92)	0.90* (0.88–0.91)	0.90* (0.89–0.92)
2–3	52924	0.16	0.91 (0.90–0.92)	0.90* (0.88–0.91)	0.90* (0.89–0.92)
4–7	67443	0.21	0.87 (0.87–0.88)	0.86* (0.86–0.87)	0.86* (0.86–0.87)
8–13	38206	0.12	0.83 (0.82–0.83)	0.81* (0.81–0.82)	0.82* (0.81–0.82)
≥14	65396	0.20	0.76 (0.75–0.76)	0.75* (0.74–0.75)	0.73* (0.73–0.74)

* Indicates significantly different from linked score C statistic (p<0.05)

**Table 6:** Discrimination by different follow up periods for the Linked score, Charlson index, & Elixhauser index.

Follow up	Number of people	follow up (person years)	Harrell’s C Statistic		
			Linked score	Charlson index	Elixhauser index
0–1	328636	1.08e+08	0.88 (0.88–0.88)	0.87* (0.87–0.87)	0.87* (0.87–0.87)
1–2	263390	1.82e+08	0.85 (0.85–0.86)	0.85* (0.85–0.85)	0.85* (0.84–0.85)
2–3	206199	2.16e+08	0.84 (0.84–0.84)	0.84* (0.83–0.84)	0.84* (0.83–0.84)
3–4	155371	2.19e+08	0.83 (0.83–0.83)	0.83* (0.82–0.83)	0.82* (0.82–0.83)
4–5	112786	1.99e+08	0.81 (0.81–0.82)	0.81* (0.81–0.81)	0.81* (0.81–0.81)

* Indicates significantly different from linked score C statistic (p<0.05)

#### Performance of the linked score in adjusting for co-morbidity in diabetes and upper gastrointestinal bleeding

The effect of an upper gastrointestinal bleeding event or a chronic diagnosis of diabetes on all-cause mortality is shown in Table 7 adjusted for the linked score, Charlson index, and Elixhauser index. The linked score had a greater effect in adjusting associated mortality for the effect of long term co-morbidity in both diabetes and upper gastrointestinal bleeding.

**Table 7:** Hazard ratios for the effect of a first upper gastrointestinal bleed on mortality.

Model with all-cause mortality as the outcome	Adjusted* hazard ratios for the effect of a coded diagnosis of upper gastrointestinal bleeding or diabetes (95% confidence interval)			
**Upper Gastrointestinal bleeding**	**60 days**		**61 days—5 years**	
Age & gender only	5.81	(5.29–6.39)	1.44	(1.31–1.57)
Charlson index from ICD 10 & Read codes	4.71	(4.28–5.17)	1.19	(1.09–1.31)
Linked score	4.59	(4.17–5.04)	1.14	(1.04–1.25)
Linked score & recent hospitalisation	4.51	(4.10–4.96)	1.12	(1.02–1.22)
**Diabetes**			**5 years**	
Age & gender only			1.38	(1.31–1.46)
Charlson index from ICD 10 & Read codes			1.18	(1.12–1.25)
Linked score			1.14	(1.08–1.20)
Linked score & recent hospitalisation			1.12	(1.06–1.18)

*Exponentiated coefficients all adjusted for age and gender (age included in baseline hazard).

## Discussion

The use of linked population based data to derive a new co-morbidity score resulted in an improvement in model fit and discrimination within a validation cohort compared to existing Charlson and Elixhauser indices. There was also a greater ability to adjust for the indirect effect of co-morbidity in the chronic disease of diabetes and the acute event of upper gastrointestinal bleeding. The improvement in discrimination was most notable for younger age groups, and was comparable across different consultation rates and follow up times.

The improved discrimination in younger age groups reflects the strength of using the linked data within an unselected general population rather than only data derived from restricted hospital admissions. This improvement partly reflects the additional disease categories identified of psychotic, neurological, and congenital conditions in addition to alcohol excess, drug misuse and traumatic burns. However, the improvements cannot be explained by just the inclusion of a greater number of diagnoses, as it also performed better than the Elixhauser index which has a similar number of diagnoses. In our previous work we have found that simply adding primary care data to secondary care data did not improve the performance of the Charlson index [17], and we confirmed that for most categories there was no difference whether the category was coded in primary or secondary care. Furthermore, there were some categories such as multiple sclerosis and genitourinary malignancy that were only relevant when coded in secondary care. However primary care categories, even when a differential effect was observed, still had a significant association with mortality.

A potential weakness of the study was including all codes in the automated model building, rather than deriving clinical disease categories from existing prior knowledge. However, this was the intention of the design of the study to allow the identification of novel groups of codes that predicted survival that might have been overlooked in pre-existing scores. The disadvantage of this method is that it might over fit a model and simply reflect bias existing within the coding rather than real clinical associations. Whilst we will have minimised this through our manual review for implausible associations, there is also a benefit in using real life patterns of coding in primary and secondary care. The resulting score therefore will have real utility in future research as it will take into account these patterns of coding where this predicts reduced survival. The bootstrapping to test for testimation bias did not suggest a large bias from over-fitting within the score development, and the internal validation we performed in a separate CPRD cohort produced similar results to the main development cohort.

As with all observational epidemiology there is still the possibility of unmeasured residual confounding, bias and random error generating spurious results. The likelihood of selection bias occurring was small as the unselected study population included all available people in the CPRD, and random error was reduced due to the large population size. Some under reporting or missing data for risk factors will be inevitable in routine data; however, by using data from both primary and secondary care we increased the sensitivity for detecting relevant co-morbidities. Misclassification was also possible, however the components of the Charlson index have previously been validated in the GPRD against case records (myocardial infarction [20, 21], heart failure [22, 23], cerebrovascular disease [24–26], dementia [27–29], respiratory disease [30–32], connective tissue disease [33–36], peptic ulcers [37], liver disease [38, 39], renal failure [40], cancer [41], leukaemia and lymphoma [42]). The HES data submissions are regularly cleaned and monitored for data quality and consistency. An in depth government audit of samples of UK hospital data confirmed accuracy approaching 90% [43]. Similarly, CPRD primary care records undergo regular quality and consistency checks and a practice’s data is only included when it is of high enough quality to be used in research (at these times the data is said to be “up to research standard”) [44]. The CPRD has been extensively validated with paper records for a wide range of diagnoses with a mean positive predictive value of 89% [45].

Another potential limitation of our study was the use of the C statistic as a measure of discrimination rather than a more ‘up to date’ method such as the Net Reclassification Index [46, 47]. We were only able to show small improvements in the C statistic within our cohort because we were making comparisons between models that all had excellent discrimination. To obtain larger improvements in the C statistic would require risk factors with unrealistically large hazard ratios [48, 49]. However, alternatives that are more sensitive to improvements in discrimination, such as the Net Reclassification Index, have been shown to be misleading, as it has been demonstrated that adding non informative data to a model can result in a beneficial Net Reclassification Index [50, 51]. Instead we show confidence intervals for C statistic comparisons and test improvements in goodness of fit. As our models were not nested we used the AIC to perform the comparisons in goodness of fit. All the improvements in C statistic were associated with significant improvements in goodness of fit as assessed by the AIC.

Previous studies have shown that using linked primary and secondary care data improved the identification of a limited number of diseases compared to using unlinked data sources. Specifically, it increased the sensitivity of identifying both acute and chronic diseases; such as diabetes [52, 53], cirrhosis [39], venous thromboembolic events [54], acute myocardial infarction [55], pneumonia [56], and acute upper gastrointestinal bleeding [18]. In contrast using only primary care data reduced the positive predictive value for acute events whilst not identifying the more severe cases found only in hospital data [18, 39, 55, 56]. Our previous work on validating the Charlson index in the linked data supported these findings, and found that primary care records did not improve the performance of the Charlson index derived from hospital records [17]. However, our study now shows that the addition of categories derived from both primary and secondary care can perform better than existing scores, demonstrating that there was discriminating information in primary care data additional to that in secondary care data. A recent paper has shown an improvement in discrimination in survival by adding socioeconomic data to the Charlson index [6]. Our linked score had a slightly improved discrimination across most socioeconomic classes (S4 Table), however the addition of socioeconomic data to the model with the linked score produced only a slight improvement in discrimination and was therefore not included in our main analysis.

## Conclusion

We have been able to demonstrate methods to derive a co-morbidity score from linked data in the unselected general population, and have shown that this derives a score that performs better across all age groups and in particular among those younger than 50 years old. The score remains robust across different consultation rates and follow up periods, and provides better adjustment for co-morbidity in both a chronic and acute disease known to have associations with mortality.

## Supporting Information

S1 Table Comparison of the distribution of the Charlson index and the linked score. Summed scores were capped at 20.
(DOCX)

S2 Table C statistic for each score stratified by quintile of deprivation (DOCX)

S3 Table ICD 10 codes for linked score categories (CSV)

S4 Table Read codes for linked score categories (CSV)
