# Supplementary material for: The Use of a Bayesian Hierarchy to Develop and Validate a Co-Morbidity Score to Predict Mortality for Linked Primary and Secondary Care Data from the NHS in England
Source: PLoS One. 2016 Oct 27;11(10):e0165507. doi: 10.1371/journal.pone.0165507 (PMC5082800; doi:10.1371/journal.pone.0165507)
Supplement: S1 Table — Summed scores were capped at 20. (DOCX) [file pone.0165507.s001.docx]

**S1 Table: Comparison of the distribution of the Charlson index and the linked score.** Summed scores were capped at 20

|  | **Charlson index** | | | | **Linked score** | | | |
| --- | --- | --- | --- | --- | --- | --- | --- | --- |
| Summed | Frequency | Percent | Hazard | 95% CI | Frequency | Percent | Hazard | 95% CI |
| score |  |  | ratios* |  |  |  | ratios* |  |
| 0 | 228,233 | 69.45 | 1 |  | 219,418 | 66.77 | 1 |  |
| 1 | 55,339 | 16.84 | 1.79 | (1.69­–1.90) | 25,543 | 7.77 | 1.47 | (1.36­–1.60) |
| 2 | 21,353 | 6.50 | 2.31 | (2.17­–2.46) | 17,223 | 5.24 | 1.77 | (1.62­–1.92) |
| 3 | 10,828 | 3.29 | 2.61 | (2.43­–2.79) | 18,180 | 5.53 | 2.03 | (1.87­–2.21) |
| 4 | 5,330 | 1.62 | 3.28 | (3.03­–3.55) | 10,661 | 3.24 | 2.51 | (2.32­–2.72) |
| 5 | 2,753 | 0.84 | 2.97 | (2.67­–3.29) | 6,814 | 2.07 | 2.68 | (2.45­–2.93) |
| 6 | 1,389 | 0.42 | 3.64 | (3.2­0–4.15) | 7,451 | 2.27 | 3.21 | (2.94­–3.51) |
| 7 | 646 | 0.20 | 4.18 | (3.52­–4.97) | 5,174 | 1.57 | 3.70 | (3.39­–4.04) |
| 8 | 1,490 | 0.45 | 7.45 | (6.68­–8.32) | 3,265 | 0.99 | 3.95 | (3.57­–4.37) |
| 9 | 657 | 0.20 | 6.97 | (6.01­–8.08) | 3,437 | 1.05 | 4.11 | (3.71­–4.55) |
| 10 | 326 | 0.10 | 8.45 | (7.03­–10.2) | 2,159 | 0.66 | 4.42 | (3.94­–4.96) |
| 11 | 155 | 0.05 | 4.78 | (3.46­–6.60) | 2,069 | 0.63 | 5.18 | (4.63­–5.80) |
| 12 | 70 | 0.02 | 4.84 | (3.08­–7.60) | 1,502 | 0.46 | 5.26 | (4.65­–5.95) |
| 13 | 29 | 0.01 | 5.76 | (3.09­–10.7) | 1,186 | 0.36 | 4.84 | (4.21­–5.57) |
| 14 | 13 | 0 | 11.5 | (4.76­–27.6) | 832 | 0.25 | 6.03 | (5.17­–7.02) |
| 15 | 11 | 0 | 7.94 | (2.98­–21.2) | 702 | 0.21 | 6.24 | (5.31­–7.34) |
| 16 | 9 | 0 | 35.7 | (17.0­–75.1) | 521 | 0.16 | 7.17 | (6.02­–8.56) |
| 17 | 2 | 0 | 0 | ­– | 511 | 0.16 | 7.48 | (6.31­–8.87) |
| 18 | 1 | 0 | 55.3 | (7.77­–392.8) | 438 | 0.13 | 8.12 | (6.79­–9.72) |
| 19 | 1 | 0 | 322.4 | (45.3­–2295.4) | 317 | 0.10 | 7.96 | (6.42­–9.87) |
| 20 | 1 | 0 | 31.8 | (4.47­–226.0) | 1,233 | 0.38 | 11.38 | (10.2­–12.7) |
| *adjusted for age, gender and recent hospitalisation | | | | | | | | |
