# Supplementary material for: The Use of a Bayesian Hierarchy to Develop and Validate a Co-Morbidity Score to Predict Mortality for Linked Primary and Secondary Care Data from the NHS in England
Source: PLoS One. 2016 Oct 27;11(10):e0165507. doi: 10.1371/journal.pone.0165507 (PMC5082800; doi:10.1371/journal.pone.0165507)
Supplement: S2 Table — (DOCX) [file pone.0165507.s002.docx]

**S2 Table: C statistic for each score stratified by quintile of deprivation**

| Quintile of deprivation* | N | (%) | Linked score | Charlson index | Elixhauser index |
| --- | --- | --- | --- | --- | --- |
| 1 | 55205.00 | 0.17 | 0.89 | 0.88 | 0.88 |
| 2 | 70271.00 | 0.21 | 0.88 | 0.88 | 0.87 |
| 3 | 68684.00 | 0.21 | 0.88 | 0.87 | 0.87 |
| 4 | 72594.00 | 0.22 | 0.88 | 0.87 | 0.87 |
| 5 | 61882.00 | 0.19 | 0.87 | 0.86 | 0.86 |

* Index of Multiple Deprivation 2007 quintile [58]. 1 = Lowest risk of deprivation.
